# Single Cell Glucose Uptake Assays: A Cautionary Tale

**DOI:** 10.20900/immunometab20200029

**Published:** 2020-08-17

**Authors:** Linda V. Sinclair, Celine Barthelemy, Doreen A. Cantrell

**Affiliations:** 1 Division of Cell Signalling and Immunology, School of Life Sciences, University of Dundee, Dundee, DD1 4HN, UK; 2 Molecular Cell Physiology, Université Libre de Bruxelles (ULB), IBMM (Biopark), Gosselies 6041, Belgium

**Keywords:** 2-NBDG, glucose uptake, T cells, T lymphocytes

## Abstract

Assays to monitor the metabolic state or nutrient uptake capacity of immune cells at a single cell level are increasingly in demand. One assay, used by many immunologists, employs 2-(*N*-(7-Nitrobenz-2-oxa-1,3-diazol-4-yl)Amino)-2-Deoxyglucose (2-NBDG), a fluorescent analogue of 2-deoxyglucose (2DG), as a substrate for glucose transporters. This molecule has been validated as a substrate for the glucose transporter Glut2 (Slc2a2) in mammalian cells but 2-NDBG selectivity for the glucose transporters expressed by T cells, Glut1 (Slc2a1) and Glut3 (Slc2a3), has never been explored. Nor has the possibility that 2-NBDG might bind to T cells that do not express glucose transporters been assessed. In this technical commentary we interrogate the specificity of 2-NBBG labelling as a readout for glucose transport in T lymphocytes. We compare flow cytometric 2-NBDG staining against well validated radiolabelled glucose transport assays in murine T cells. Our data show there can be a large discordance between glucose transport capacity and 2-NBDG labelling in T cells. We also find that 2-NBDG uptake into murine T cells is not inhibited by competitive substrates or facilitative glucose transporter inhibitors, nor can 2-NBDG competitively block glucose uptake in T cells. Collectively, these data argue that 2-NBDG uptake alone is not a reliable tool for the assessment of cellular glucose transport capacity.

## Introduction

In recent years, there has been a focus on intracellular metabolic pathways, metabolites and their dynamic changes in immune cells. Alongside this there is greater awareness of how nutrient availability, and the ability of immune cells to uptake nutrients, impacts and regulates immune responses. In this context, proteomic studies have shown the expression of nutrient transporters in T cells is highly regulated by antigen and cytokines [1–3]. There have also been many studies using radiolabelled substrates for both quantitative and kinetic assays of how immune cells modulate nutrient transport. For example, radiolabelled 2-deoxyglucose has been used to quantify changes in glucose transport in antigen and cytokine stimulated T cells. Similarly, radiolabelled amino acids have been used to quantify amino acid transport in immune activated T cells [3–10].

The use of radiolabelled glucose and amino acids has generated fundamental information regarding nutrient transporter dynamics, specificity and affinity in lymphocytes. However, radiolabelled tracer assays have their limitations; apart from specific equipment and training being required, radiolabelled uptake assays must be performed on whole populations and do not have the capacity to resolve changes happening in subpopulations or single cells present in a complex mixture of cells. Increasingly, it is recognised that there is a need for single cell assays that can monitor nutrient transport to further investigations into how immune cells change nutrient utilisation during immune responses. Addressing this, there is a recently described flow cytometry-based assay for System L mediated amino acid uptake [7]. This assay uses a fluorescent substrate to measure the amino acid transport capacity of single cells present in complex mixed populations or tissues[7,11,12]. In this context, there is also a widely used flow cytometry assay that uses 2-(*N*-(7-Nitrobenz-2-oxa-1,3-diazol-4-yl)Amino)-2-Deoxyglucose (2-NBDG), a fluorescent analogue of 2-deoxyglucose (2DG) to monitor glucose transporter expression by single cells. In generating fluorescent glucose analogues, the nitrobenzoxydiazoamino group (NBDG) replaces a hydroxyl group (-OH) of a glucose molecule. In 2-NBDG, it is the -OH of carbon 2 that is replaced, whereas in 6-NBDG, the -OH of carbon 6 is replaced with NBDG. This structural alteration perturbs the interaction between substrate and transporter, as modelled and described for 6-NBDG having much higher binding affinity to Glut1, compared with glucose, yet far slower transmembrane transport in astrocytes [13]. Initial characterisation and validation of 2-NBDG as a substrate for glucose transporters was done in E.coli and in these bacteria, 2-NBDG uptake by cell was effectively competed with d-glucose, partially blocked by galactose and fructose, but not blocked by l-glucose or sucrose [14]. Specifically, it has been shown that 2-NBDG uptake in bacteria is mediated by a mannose or a glucose/mannose transporter system [15]. Subsequent studies have looked to see if 2-NBDG could be used to monitor glucose transport in mammalian cells [16]. This work rigorously characterised Slc2a2 (Glut2) mediated 2-NBDG transport in Cos1 cells and in MIN6 pancreatic beta cells. Importantly, the uptake of 2-NBDG in these cells was shown to be highly selective; it was competitively inhibited by excess d-glucose and blocked in the presence of cytochalasin B, a glucose transporter inhibitor [16]. In kidney cells 2-NBDG uptake was shown to be mediated by a sodium-glucose linked transporter (SGLT), which is blocked by phlorizin with residual sodium independent transport blocked by cytochalasin B [17]. 2-NBDG uptake has since been used extensively as a readout for glucose uptake in a broad range of mammalian cell types including T lymphocytes [18–24]. Moreover, high 2-NBDG staining often correlates with glycolytic metabolic activity in activated CD8 T cells [25–28].

**Figure 1:**
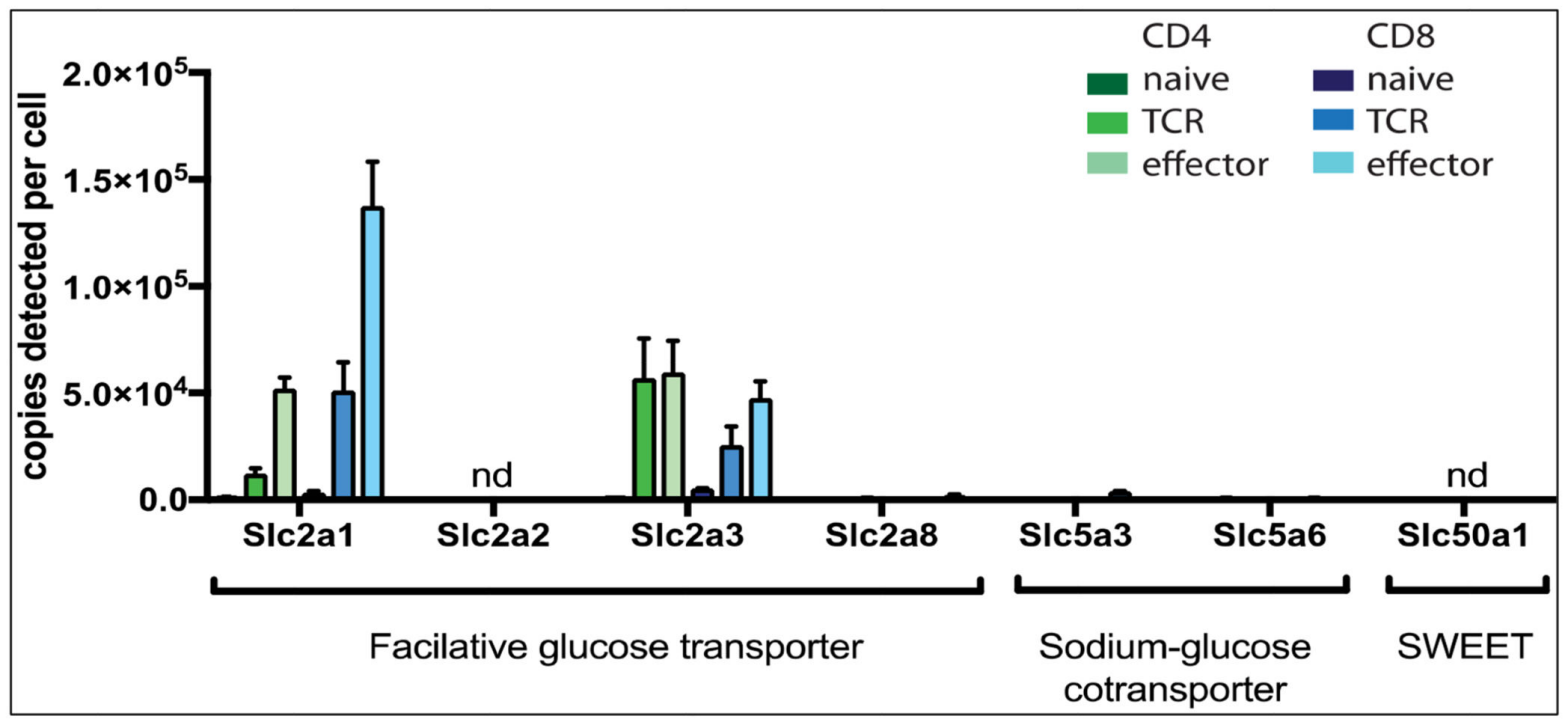
Expression profile of glucose transporters in T cells. Protein copy numbers of glucose transporters detected in naïve, 24 h activated (TCR) and effector murine CD4^+^ (TH1) and CD8^+^ (CTL) T cells. Data are from previously published proteomics data sets [2]. Mean protein copy numbers are estimated using the proteomic ruler protocol [16]. Error bars are mean+/− s.d of 3 biological replicates. nd = not detected.

However, there has been very little validation of how well 2-NBDG performs as a glucose transporter substrate in T cells and the efficacy of this assay compared to the well validated ^3^H-2DG uptake assay has not been addressed. This is pertinent as it is essential to understand the extent to which 2-NBDG binds non-selectively to different lymphocyte populations and whether changes in T cell activation and possibly autofluorescence impact the sensitivity of the 2-NBDG uptake assay. It is also critical to assess the selectivity of 2-NBDG for the glucose transporters predominantly expressed by T cells; Slc2a1(Glut 1) and Slc2a3 (Glut 3). To determine which glucose transporters are expressed by murine T cells, we have interrogated published quantitative deep proteomic T cell data sets [2]. The data in Figure 1 show that T lymphocytes do not express Slc2a2 (Glut 2) or other facilitative glucose transporter (SLC2) family members, nor do they express sodium-glucose cotransporter (SLC5) or other sugar transporters such as the SLC50 family (SWEETs). Accordingly, we compared the sensitivity of the fluorescent 2-NBDG uptake and ^3^H-2DG assay in different T cell populations including naive T cells before and after antigen receptor activation, effector CD8^+^ cytolytic T cells (CTL) and thymocyte subpopulations.

## Discrepancies between 2-NBDG and ^3^H-2DG Transport in T Cells

**Figure 2:**
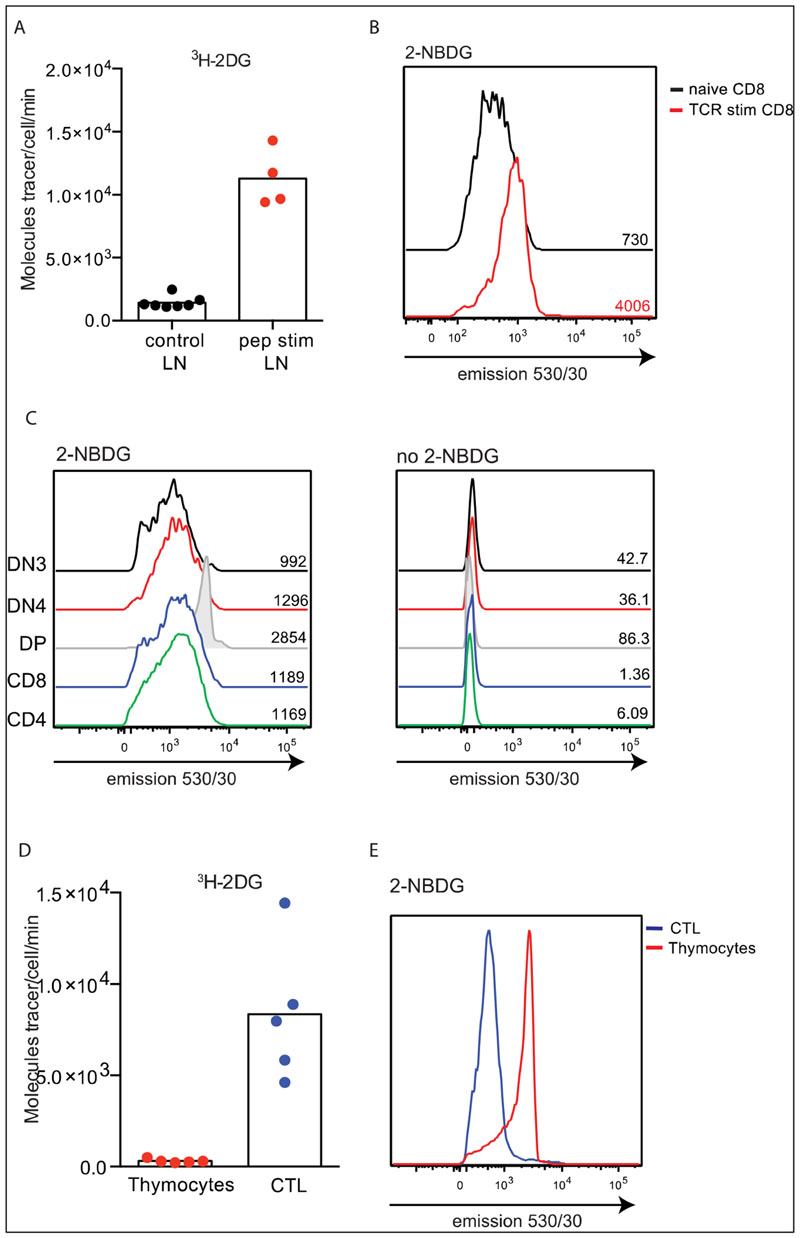
Comparison of ^3^H-2DG uptake and 2-NBDG labelling in T cells. (**A**) Uptake of ^3^H-2DG in purified P14 CD8^+^ T cells +/− TCR activation using cognate peptide (gp33) for 18 h. (**B**) Flow cytometry histograms show 2-NBDG labelling (50 μM, 10 min, 37 °C) of P14 CD8^+^ T cells +/− TCR activation using cognate peptide (gp33) for 18 h. 2-NBDG labelling is detected using bandwidth covering emission at 530/30 nm. (**C**) Histograms show 530 nm emission in thymocyte populations with (left) or without (right) 2-NBDG labelling (50 μM, 10 min, 37 °C). (**D**) Uptake of ^3^H-2DG in thymocytes (total thymus) or effector CTL. **(E)** Histograms show 2-NBDG labelling (detected at 530 nm; 50 μM, 10 min, 37 °C) of thymocytes (total thymus) or effector CTL. (A, D; radiolabelled uptakes performed in triplicate. Points indicate individual biological replicates. B, C, E; Mean fluorescent intensities (MFI) values are indicated on the histograms, data are representative from minimum 3 biological replicates.).

Figure 2A shows the quantification of glucose transport in naive and antigen receptor activated CD8^+^ T cells using the ^3^H-2DG assay. These data show a 10-fold increase in glucose transport in antigen activated T cells compared to naive T cells. Figure 2B compares 2-NBDG binding to naive and antigen receptor activated T cells and shows that 2-NBDG binding to T cells is increased following immune activation; and activated T cells have a 5-fold higher level of 2-NBDG binding than naive T cells. Glucose transporter expression, as determined by proteomics data analysis, shows 3000 copies of Slc2a1 and 5000 copies of Slc2a3 expressed in naive T cells versus 51,000 Slc2a1 and 25,000 Slc2a3 copies in antigen activated T cells: an overall 10-fold increase in glucose transporter expression (Figure 1). Thus, both the ^3^H-2DG and the 2-NBDG labelling data appear to correlate well with relative levels of expression of glucose transporters in these T cell populations.

Next we measured the effectiveness of the 2-NBDG labelling assay in thymocytes. Radiolabelled glucose transport assays have shown that murine T cell progenitors in the DN3 stage of thymocyte development (CD4^−^CD8^−^CD44^−^CD25^+^) have low levels of glucose transport but once these cells undergo TCR β-selection and transit to DN4 thymocytes they upregulate glucose transport to fuel rapid self-renewal and differentiation into CD4^+^CD8^+^double-positive (DP) thymocytes. DPs return to a state of quiescence and have very low levels of glucose transport [10,29]. This pattern of high glucose uptake and Glut1 expression post TCR β-selection is also closely paralleled in human thymocytes [30]. Figure 2C shows 2-NBDG staining in thymocytes and reveals that the highest levels of 2-NBDG labelling are present in DP thymocytes, and this is not due to heightened autofluorescence in DP cells. Thus in the thymus there is poor correlation between 2-NBDG labelling and glucose transport capacity. This discrepancy between 2-NBDG labelling and ^3^H-2DG uptake is further underlined when thymocyte uptake is compared with effector T cells. Effector CD8 T cells have high levels of ^3^H-2DG uptake compared with almost undetectable ^3^H-2DG uptake by thymocytes, which are 80–90% DP cells (Figure 2D). In comparison, flow cytometric analysis of 2-NBDG labelling shows very high levels of 2-NBDG staining on DP thymocytes, even higher than that seen in effector CD8 T cells (Figure 2E).

## 2-NBDG Uptake is not Sensitive to Glucose Transporter Inhibition

**Figure 3:**
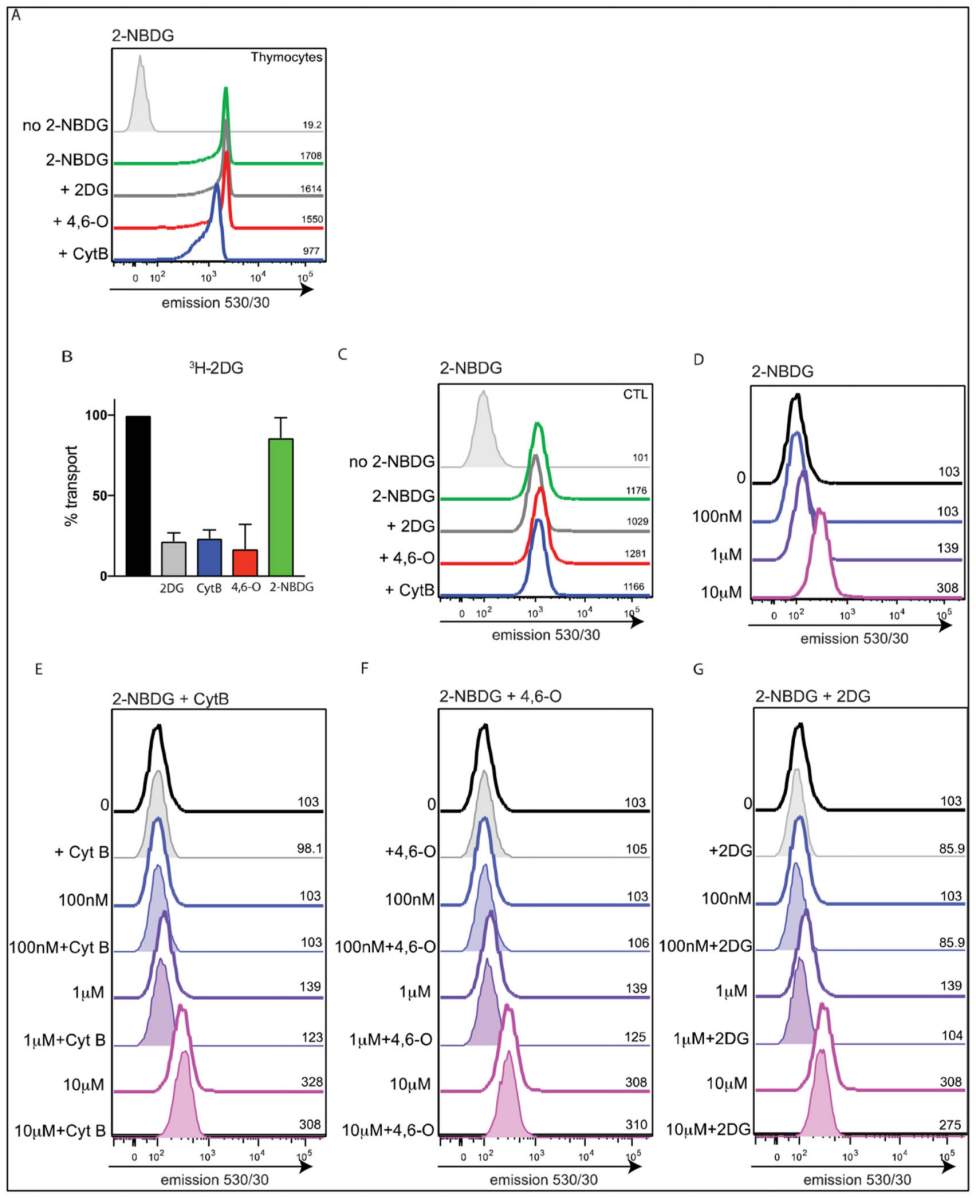
Glucose transporter inhibition does not block 2-NBDG labelling in T cells. **(A)** Histograms show 2-NBDG labelling (detected at 530 nm) of thymocytes (total thymus) in the presence or absence of 2DG (50 mM), 4,6-*O*-ethylidene-α-d-glucose (4,6-*O*; 20 mM) or cytochalasin B (CytB; 10 μM). **(B)** Percentage of ^3^H-2DG uptake in effector CTL inhibited by 2DG (5 mM), 4,6-*O*-ethylidene-α-d-glucose (4,6-*O*; 20 mM), cytochalasin B (CytB;10 μM) or 2-NBDG (5 mM). **(C)** Histograms show 2-NBDG labelling (detected at 530 nm) of effector CTL in the presence or absence of 2DG (50 mM), 4,6-*O*-ethylidene-α-d-glucose (4,6-*O*; 20 mM) or cytochalasin B (CytB;10 μM). **(D)** Histograms show 2-NBDG labelling (detected at 530 nm) of effector CTL after incubation with 100 nM, 1 μM or 10 μM 2-NBDG for 30 min. **(E–G)** Histograms show 2-NBDG labelling (detected at 530 nm) of effector CTL after incubation with 100 nM, 1 μM or 10 μM 2-NBDG for 30 min in the presence or absence of fixed concentrations of glucose transport inhibitors: CytB (10 μM; E), 4,6-*O* (20 mM; F) or 2DG (50 mM; G). (A, C–G; MFI values are indicated on the histograms, data are representative from 3 independent experiments. b; radiolabelled uptakes performed in triplicate).

In subsequent experiments we used glucose transporter inhibitors or transporter substrate competition to interrogate the specificity of 2-NBDG uptake in T cells. Cytochalasin B binds strongly to the internal face of facilitative glucose transporters (Slc2a1, Slc2a2, Slc2a3 and Slc2a4) and blocks substrate import. 4,6-*O*-ethylidene-α-d-glucose (4,6-*O*) binds at an exofacial (outer) site and efficiently blocks substrate binding to the transporter [31]. Non-radiolabelled 2-deoxyglucose (2DG) was used as a direct substrate competition for the radiolabelled ^3^H-2DG uptake; this competes with glucose for binding and transport. The data show that neither 4,6-*O* treatment, cold competition with excess 2DG or glucose decreased the high levels of 2-NBDG labelling seen in thymocytes (Figure 3A). Nor did cytochalasin B treatment block 2-NBDG staining in the thymocytes, although there was a consistent small reduction in uptake (Figure 3A). In comparison, Figure 3B shows that ^3^H-2DG uptake into effector T cells is efficiently blocked by cytochalasin B, 4,6-*O* and 2-DG. Whereas, 2-NBDG staining of effector T cells is not inhibited by cytochalasin B, 4,6-*O* or 2-DG (Figure 3C). In further experiments we assessed the impact of increasing the ratio of competitor/inhibitor relative to 2-NBDG in the uptake assays. There was no detectable 2-NBDG staining below 1μM 2-NBDG (Figure 3D). At no point did glucose transporter inhibition with either cytochalasin B (Figure 3E) or 4,6-*O* (Figure 3F) block the 2-NBDG signal. There was a very small shift in CTL fluorescence in the presence of 50 mM 2-DG. This small reduction in fluorescence was not, however, specific to 2-NBDG uptake as this was present in unstained cells as well (Figure 3G). An explanation for this effect of 2DG on cell autofluorescence is that 2DG, which is known to interfere with glucose metabolism and glycolysis in T cells, could impact/perturb the NAD(P)H autofluorescence normally seen in highly glycolytic cells. This highlights another point of caution when interpreting subtle changes in fluorescence whilst using dyes that are detected in the region of cellular autofluorescence: you need to be certain that these differences are due to the amount of dye and not due to altered cellular autofluorescence. Collectively these data show a disconnect between radiolabelled 2DG transport and 2-NBDG transport in T cells and argue that glucose transport and 2-NBDG uptake are not mediated by a common transporter. Further evidence for this concept comes from experiments where 2-NBDG was not able to compete ^3^H-2DG uptake by activated T cells (Figure 3B).

## Discussion

In summary, the present data show that 2-NBDG labelling can correlate with metabolically active cells; e.g., activated T cells have higher 2-NBDG staining than naïve T cells. However, this is not always the case and we have observed the highest levels of 2-NBDG staining in non-metabolically active CD4^+^CD8^+^double-positive (DP) thymocytes that have very low levels of glucose transport. Moreover, in order for a substrate to be a reporter for glucose transporter activity, it should conform to relevant transporter dynamics. It is of note to recognise that initial testing and characterisation of 2-NBDG in mammalian cells used clear parameters to validate transport specificity; cytochalasin B treatment or competitive substrate assays thus led to inhibition of 2-NBDG transport [13,16]. Herein we have applied these parameters to 2-NBDG labelling of T cells and the data show clearly that 2-NBDG staining of murine thymocytes and effector CD8^+^ T cells does not follow simple glucose transporter principles: it is not out-competed by substrates, nor is it blocked by transporter inhibition.

It is noteworthy that the discrepancy between NBDG analogue uptake and bonafide 2DG or glucose uptake has been previously noted [13,32]. Barros et al used mathematical modelling to address the disparity between the rates of glucose and 6-NBDG uptake in astrocytes as well as the insensitivity of 6-NBDG uptake to either glucose competition or cytochalasin B inhibition [13]. Their proposed model describes that 6-NBDG binds to GLUT1 with 300 times higher affinity than glucose, but is not efficiently translocated, remaining on the exofacial surface of the cell. Thus, inhibitors which bind the exofacial aspect of glucose transporters would be predicted to be more effective at blocking NBDG import/labelling than endofacial inhibitors. Our data presented herein shows that whilst exofacial inhibition using 4,6-*O* does block radiolabelled 2DG uptake, it does not inhibit 2-NBDG accumulation in T cells. This and the fact that 2-NBDG cannot competitively prevent glucose transport by activated T cells brings into question the validity of 2-NBDG as a tool to monitor glucose transport. Assays for nutrient transport have to incorporate relevant transporter controls, ensuring specificity and selectivity in the system. The lack of appropriate controls to verify the specificity of the 2-NBDG assay is a problem. Moreover, the high binding of 2-NBDG to thymocytes highlights the potential for 2-NBDG assays to misinform about cellular glucose transport activity. If thymocytes can have high uptake of 2-NBDG with no discernible capacity to transport glucose, then how many other leucocyte populations show a similar disconnect? These data highlight that 2-NBDG uptake assays should be interpreted cautiously and conclusions about cellular glucose transport capacity based on NBDG modified glucose uptake should only be made when the experiments include appropriate controls.

## Materials and Methods

### Mice and Cells

C57BL/6 (wild-type, WT) and P14 TCR [33] transgenic mice were bred and maintained in the WTB/RUTG, University of Dundee in compliance with UK Home Office Animals (Scientific Procedures) Act 1986 guidelines. All studies were performed on project license PPL60/4488 (Granted: 2013–March-21) or P4BD0CE74 (Granted: 2018-March-21), approved by the University of Dundee Welfare and Ethical Use of Animals Committee and in compliance with UK Home Office Animals (Scientific Procedures) Act 1986 guidelines.

For naïve and TCR activated cells, lymph nodes from P14 TCR transgenic mice were removed and disaggregated. For TCR activated cells, lymph node suspensions were stimulated with cognate antigenic peptide (glycoprotein amino acids 33–41 (GP33); 100 ng/mL) in the presence of cytokines IL12 (10 ng/mL; RnD Systems, Abingdon, UK) and 20 ng/mL IL2 (20 ng/mL; Proleukin, Novartis, London, UK). Cells were cultured in RPMI 1640 containing l-glutamine (cat# 11875093, Gibco, ThermoFisher Scientific, UK), 10% FBS (cat#26140087, Gibco, ThermoFisher Scientific, UK), 50 μM β-mercaptoethanol (cat#31350010, β-ME, Gibco, Sigma-Aldrich, UK) and penicillin/streptomycin (cat#15070063, Gibco, ThermoFisher Scientific, UK). Prior to radiolabelled uptake, CD8^+^ T cells were isolated using a magnetic bead negative selection kit (cat#19853, EasySep, STEMCELL Technologies, Cambridge, UK). To generate effector CTL, spleens were extracted from P14 mice and mashed in red blood cell lysis buffer before being suspended in RPMI media supplemented with GP33 peptide (100 ng/mL) and IL-2 (20 ng/mL) and IL-12 (10 ng/mL) for 48 h. Subsequently, cells were washed out of activation media and then cultured for a further 3 days in media supplemented with IL-2 (20 ng/mL) and IL-12 (2 ng/mL), cells were maintained at 2 × 10^5^ per mL. Cells were incubated at 37 °C with 5% CO_2_ throughout.

To isolate thymocytes, thymi from 6–8 week old WT mice were removed and disaggregated. Cells were resuspended in RPMI 1640 containing l-glutamine, 10% FBS, 50 μM β-ME and penicillin/streptomycin.

### Radiolabelled 2-Deoxyglucose Uptake

Briefly, [^3^H]-2-deoxyglucose (^3^H-2DG, cat#NET328A; Perkin Elmer, Beaconsfield, UK) uptake was carried out using 1 × 10^6^ cells resuspended in 0.4 mL uptake medium. Each uptake for a biological replicate is performed in triplicate. ^3^H-2DG uptake was carried out in glucose free RPMI (cat#11879020, ThermoFisher Scientific, UK) containing ^3^H-2DG (1 μci/ml). 4 min uptake assays were carried out layered over 0.5 mL of 1:1 silicone oil (Dow Corning 550 (BDH silicone products; specific density, 1.07 g/mL: Cat#175633, Sigma-Aldrich, UK):dibutyl phthalate (Cat#524980, Sigma-Aldrich, UK). Cells were pelleted below the oil, the aqueous supernatant solution, followed by the silicone oil/dibutyl phthalate mixture was aspirated, and the cell pellet underneath resuspended in 200 μL NaOH (1 M) and β-radioactivity measured by liquid scintillation counting in a Beckman LS 6500 Multi-Purpose Scintillation Counter (scintillant Optiphase HiSafe 3, cat#1200.437; PerkinElmer, Beaconsfield, UK). Where indicated, 5 mM 2-deoxyglucose (2DG, cat#D6134; Sigma-Aldrich, UK), 5 mM 2-NBDG (cat#N13195, ThermoFisher Scientific, UK), 20 mM 4,6-*O*-ethylidene-α-d-glucose (4,6-*O*, cat#E32754; Sigma-Aldrich, UK) or 10 μM cytochalasin B (CytB, cat#C6762; Sigma-Aldrich, UK), were used respectively to inhibit radiolabelled ligand uptake.

### Flow Cytometry

#### 2-NBDG labelling

2-(*N*-(7-Nitrobenz-2-oxa-1,3-diazol-4-yl)Amino)-2-Deoxyglucose (2-NBDG, cat#N13195; ThermoFisher Scientific, UK) was stored as a 10 mM stock solution. Unless otherwise indicated, the data presented show T cell 2-NBDG labelling performed at a final concentration of 50 μM in glucose free RPMI at 37 °C for 10 min. (Longer time courses, with higher and lower concentration have been performed, data not shown.) Where indicated, 50 mM 2DG, 20 mM 4,6-*O* or 10 μM CytB were used respectively to inhibit glucose transporter function.

#### Thymocyte staining and gating strategy

Cell surface staining of isolated thymocytes was performed with CD4-Alexa 700 (RM4-5, cat#116022) CD8a-BV421 (53–67, cat#100737), CD25-PECy7 (PC61, cat#102016) and CD44-BV510 (IM7, cat#103043). Antibodies from BioLegend, UK. DN3 cells were identified as CD4^−^ CD8^−^ CD44^−^ CD25^+^; DN4 cells were identified as CD4^−^ CD8^-^ CD44^−^ CD25^−^; DP cells were identified as CD4^+^ CD8^+^; CD4^+^ cells were identified as CD4^+^ CD8^−^ and CD8^+^ cells were identified as CD4^−^ CD8^+^.

Data were acquired on a LSR Fortessa II with DIVA software or a FACSVerse flow cytometer with FACSuite software (BD Biosciences-Europe, Oxford, UK) and analyzed using FlowJo software (for Mac, version 9 and 10, Treestar; BD Biosciences-Europe, Oxford, UK).

## References

[1] Hukelmann JL, Anderson KE, Sinclair LV, Grzes KM, Murillo AB, Hawkins PT (2016). The cytotoxic T cell proteome and its shaping by the kinase mTOR. Nat Immunol.

[2] Howden AJM, Hukelmann JL, Brenes A, Spinelli L, Sinclair LV, Lamond AI (2019). Quantitative analysis of T cell proteomes and environmental sensors during T cell differentiation. Nat Immunol.

[3] Sinclair LV, Howden AJ, Brenes A, Spinelli L, Hukelmann JL, Macintyre AN (2019). Antigen receptor control of methionine metabolism in T cells. Elife.

[4] Jacobs SR, Michalek RD, Rathmell JC (2010). IL-7 Is Essential for Homeostatic Control of T Cell Metabolism In Vivo. J Immunol.

[5] Frauwirth KA, Riley JL, Harris MH, Parry RV, Rathmell JC, Plas DR (2002). The CD28 signaling pathway regulates glucose metabolism. Immunity.

[6] Carr EL, Kelman A, Wu GS, Gopaul R, Senkevitch E, Aghvanyan A (2010). Glutamine Uptake and Metabolism Are Coordinately Regulated by ERK/MAPK during T Lymphocyte Activation. J Immunol.

[7] Sinclair LV, Neyens D, Ramsay G, Taylor PM, Cantrell DA (2018). Single cell analysis of kynurenine and System L amino acid transport in T cells. Nat Commun.

[8] Sinclair LV, Rolf J, Emslie E, Shi Y-B, Taylor PM, Cantrell DA (2013). Control of amino-acid transport by antigen receptors coordinates the metabolic reprogramming essential for T cell differentiation. Nat Immunol.

[9] Grzes KM, Swamy M, Hukelmann JL, Emslie E, Sinclair L, Cantrell DA (2017). Control of amino acid transport coordinates metabolic reprogramming in T-cell malignancy. Leukemia.

[10] Swamy M, Pathak S, Grzes KM, Damerow S, Sinclair LV, van Aalten DMF (2016). Glucose and glutamine fuel protein *O*-GlcNAcylation to control T cell self-renewal and malignancy. Nat Immunol.

[11] Loftus RM, Assmann N, Kedia-Mehta N, O’Brien KL, Garcia A, Gillespie C (2018). Amino acid-dependent cMyc expression is essential for NK cell metabolic and functional responses in mice. Nat Commun.

[12] O’Brien A, Loftus RM, Pisarska MM, Tobin LM, Bergin R, Wood NAW (2019). Obesity Reduces mTORC1 Activity in Mucosal-Associated Invariant T Cells, Driving Defective Metabolic and Functional Responses. J Immunol.

[13] Barros LF, Bittner CX, Loaiza A, Ruminot I, Larenas V, Moldenhauer H (2009). Kinetic validation of 6-NBDG as a probe for the glucose transporter GLUT1 in astrocytes. J Neurochem.

[14] Yoshioka K, Takahashi H, Homma T, Saito M, Oh KB, Nemoto Y (1996). A novel fluorescent derivative of glucose applicable to the assessment of glucose uptake activity of Escherichia coli. Biochim Biophys Acta.

[15] Tao J, Diaz RK, Teixeira CRV, Hackmann TJ (2016). Transport of a Fluorescent Analogue of Glucose (2-NBDG) versus Radiolabeled Sugars by Rumen Bacteria and Escherichia coli. Biochemistry.

[16] Yamada K, Nakata M, Horimoto N, Saito M, Matsuoka H, Inagaki N (2000). Measurement of glucose uptake and intracellular calcium concentration in single, living pancreatic beta-cells. J Biol Chem.

[17] Blodgett AB, Kothinti RK, Kamyshko I, Petering DH, Kumar S, Tabatabai NM (2011). A fluorescence method for measurement of glucose transport in kidney cells. Diabetes Technol Ther.

[18] Funabashi H, Ogino S, Saito M, Matsuoka H (2012). Utilization of Fluorescent Glucose Analog 2-NBDG as a Metabolic Indicator for FACS Analysis during ES Cell Differentiation. Electrochemistry.

[19] Everts B, Amiel E, Huang SC-C, Smith AM, Chang C-H, Lam WY (2014). TLR-driven early glycolytic reprogramming via the kinases TBK1-IKKε supports the anabolic demands of dendritic cell activation. Nat Immunol.

[20] Dupuy F, Griss T, Blagih J, Bridon GE, Avizonis D, Ling C (2013). LKB1 is a central regulator of tumor initiation and pro-growth metabolism in ErbB2-mediated breast cancer. Cancer Metab.

[21] Blagih J, Coulombe F, Vincent EE, Dupuy F, Galicia-Vázquez G, Yurchenko E (2015). The Energy Sensor AMPK Regulates T Cell Metabolic Adaptation and Effector Responses In Vivo. Immunity.

[22] Burgener A-V, Bantug GR, Meyer BJ, Higgins R, Ghosh A, Bignucolo O (2019). SDHA gain-of-function engages inflammatory mitochondrial retrograde signaling via KEAP1-Nrf2. Nat Immunol.

[23] Pacella I, Procaccini C, Focaccetti C, Miacci S, Timperi E, Faicchia D (2018). Fatty acid metabolism complements glycolysis in the selective regulatory T cell expansion during tumor growth. Proc Natl Acad Sci U S A.

[24] Donnelly RP, Loftus RM, Keating SE, Liou KT, Biron CA, Gardiner CM (2014). mTORC1-dependent metabolic reprogramming is a prerequisite for NK cell effector function. J Immunol.

[25] Sukumar M, Liu J, Ji Y, Subramanian M, Crompton JG, Yu Z (2013). Inhibiting glycolytic metabolism enhances CD8^+^ T cell memory and antitumor function. J Clin Invest.

[26] Chang C-H, Qiu J, O’Sullivan D, Buck MD, Noguchi T, Curtis JD (2015). Metabolic Competition in the Tumor Microenvironment Is a Driver of Cancer Progression. Cell.

[27] Scharping NE, Menk AV, Moreci RS, Whetstone RD, Dadey RE, Watkins SC (2016). The Tumor Microenvironment Represses T Cell Mitochondrial Biogenesis to Drive Intratumoral T Cell Metabolic Insufficiency and Dysfunction. Immunity.

[28] Siska PJ, van der Windt GJW, Kishton RJ, Cohen S, Eisner W, MacIver NJ (2016). Suppression of Glut1 and Glucose Metabolism by Decreased Akt/mTORC1 Signaling Drives T Cell Impairment in B Cell Leukemia. J Immunol.

[29] Ciofani M, Zúñiga-Pflücker JC (2005). Notch promotes survival of pre-T cells at the beta-selection checkpoint by regulating cellular metabolism. Nat Immunol.

[30] Swainson L, Kinet S, Manel N, Battini J-L, Sitbon M, Taylor N (2005). Glucose transporter 1 expression identifies a population of cycling CD4^+^ CD8^+^ human thymocytes with high CXCR4-induced chemotaxis. Proc Natl Acad Sci U S A.

[31] Holman GD (2018). Chemical biology probes of mammalian GLUT structure and function. Biochem J.

[32] Kim WH, Lee J, Jung D-W, Williams DR (2012). Visualizing sweetness: increasingly diverse applications for fluorescent-tagged glucose bioprobes and their recent structural modifications. Sensors.

[33] Pircher H, Bürki K, Lang R, Hengartner H, Zinkernagel RM (1989). Tolerance induction in double specific T-cell receptor transgenic mice varies with antigen. Nature.

